# Screening for anti-HCV antibodies in a cohort of people living with HIV at “Zia Angelina” Medical Centre, Kampala, Uganda

**DOI:** 10.11604/pamj.2025.51.85.47997

**Published:** 2025-08-05

**Authors:** Francesco De Maria, Fernando Rico González, Alice Joy Namuwonge, Riccardo Serraino, Carlo Torti

**Affiliations:** 1 Dipartimento di Scienze Mediche e Chirurgiche, Università “Magna Graecia”, Catanzaro, Italy,; 2 Zia Angelina Health Centre of Kampala, Kampala, Uganda,; 3 Dipartimento di Scienze Mediche e Chirurgiche, Università “Magna Graecia”, Catanzaro, Italy,; 4 UOC Malattie Infettive, Dipartimento Scienze Mediche e Chirurgiche, Fondazione Policlinico Universitario Agostino Gemelli IRCCS, Rome, Italy,; 5 Sezione Malattie Infettive, Dipartimento di Sicurezza e Bioetica, Università Cattolica del Sacro Cuore, Rome, Italy

**Keywords:** Anti-HCV antibodies, people living with HIV, Uganda

## To the editors of the Pan African Medical Journal

People living with HIV (PLWH) in low-income countries face a high burden of communicable diseases, with co-infections such as the Hepatitis C virus (HCV) remaining a major concern. The consequences of HCV are more severe in individuals with HIV, and yet, in countries like Uganda, routine screening for HCV among PLWH is not standard. Our pilot study aimed to estimate the prevalence of HIV-HCV co-infection in this population at the Zia Angelina Medical Centre, a category III health facility in Namugongo, near Kampala [1,2]. Between January and October 2021, 300 HIV-positive individuals were called for anti-HCV antibody testing using a fourth-generation rapid test (LABOQUIK). Of these, 279 (93%) presented for screening. Only two patients (0.7%), both female, tested positive for anti-HCV antibodies. Among them, one showed abnormal liver enzyme values. The limited number of confirmed positive cases suggests a low prevalence of HCV among PLWH in this Ugandan setting [3].

While our findings align with previous reports suggesting stable or declining HCV trends in this population, several limitations must be acknowledged. The reliance on serological testing without confirmatory HCV RNA assays may underestimate prevalence due to false negatives during the window period or interference from co-endemic infections such as malaria [4,5]. Moreover, our sample size, although higher than similar studies in comparable settings, limits generalizability [6]. Despite these limitations, this study contributes valuable local data to the sparse literature on HIV-HCV co-infection in Uganda. It underscores the feasibility of implementing integrated screening models in outpatient HIV care, even in resource-constrained environments. Given the WHO's target of eliminating viral hepatitis as a public health threat by 2030, our experience highlights the urgent need to expand routine HCV screening among PLWH using reliable diagnostic algorithms, including RNA testing where feasible [7-10].

## Conclusion

This study confirms the feasibility and relevance of combined screening programs for co-infections in HIV-positive populations in Uganda. Despite its limitations, the study provides updated local data that can support public health efforts toward viral hepatitis elimination.

## References

[1] World Health Organization (2020). Global Health Observatory: The Top 10 causes of death.

[2] Platt L, Easterbrook P, Gower E, McDonald B, Sabin K, McGowan C (2016). Prevalence and burden of HCV co-infection in people living with HIV: a global systematic review and meta-analysis. Lancet Infect Dis.

[3] Baseke J, Musenero M, Mayanja-Kizza H (2015). Prevalence of hepatitis B and C and relationship to liver damage in HIV infected patients attending Joint Clinical Research Centre Clinic (JCRC), Kampala, Uganda. Afr Health Sci.

[4] Solomon SS, Wagner-Cardoso S, Smeaton L, Sowah LA, Wimbish C, Robbins G (2022). A minimal monitoring approach for the treatment of hepatitis C virus infection (ACTG A5360 [MINMON]): a phase 4, open-label, single-arm trial. Lancet Gastroenterol Hepatol.

[5] Hladik W, Kataaha P, Mermin J, Purdy M, Otekat G, Lackritz E (2006). Prevalence and screening costs of hepatitis C virus among Ugandan blood donors, 2000. Trop Med Int Health.

[6] Seremba E, Ocama P, Opio CK, Kagimu M, Thomas DL, Yuan HJ (2010). Poor performance of hepatitis C antibody tests in hospital patients in Uganda. J Med Virol.

[7] Mayanja E, Luboobi LS, Kasozi J, Nsubuga RN (2020). Mathematical modelling of HIV-HCV coinfection dynamics in absence of therapy. J Appl Math.

[8] World Health Organization (2016). Combating hepatitis B and C to reach elimination by 2030: Advocacy brief. WHO.

[9] Rao VB, Johari N, du Cros P, Messina J, Ford N, Cooke GS (2015). Hepatitis C seroprevalence and HIV co-infection in sub-Saharan Africa: a systematic review and meta-analysis. Lancet Infect Dis.

[10] Picchio CA, Nicolàs A, Ayemfouo Fofou IV, Kasone V, Guewo-Fokeng M, Tagny CT (2024). Acceptability and feasibility of the plasma separation card for an integrated model of care for HBV and HCV screening among people attending HIV clinics in Cameroon and Uganda. J Epidemiol Glob Health.

